# Recurrence of Multiple Gastrointestinal Angioectasias Despite Treatment with Argon Plasma Coagulation Requiring Thalidomide Treatment in a Patient with Cirrhosis: A Rare Case Report

**DOI:** 10.7759/cureus.4196

**Published:** 2019-03-06

**Authors:** Azad Mojahedi, Amrendra Mandal, Paritosh Kafle, Shambhu Bhagat, Vijay Gayam

**Affiliations:** 1 Internal Medicine, Interfaith Medical Centre, Brooklyn, USA; 2 Internal Medicine, Interfaith Medical Center, Brooklyn, USA

**Keywords:** angiodysplasia, upper gastrointestinal bleeding, thalidomide

## Abstract

Gastrointestinal (GI) angioectasia is an important cause of acute GI bleed, particularly in the elderly; however, GI angioectasia is an uncommon cause of upper GI bleeding related to cirrhosis. With the increasing incidence of liver cirrhosis and recent improvements in the treatment of advanced cirrhosis, this condition may become more common and should be considered a differential diagnosis in patients with cirrhosis who present with occult or overt GI blood loss.

We present the case of a 66-year-old man with liver cirrhosis admitted with acute upper GI bleeding that was found to have antral and duodenal angioectasia during esophagogastroduodenoscopy (EDG). Argon plasma coagulation (APC), which is considered the gold standard treatment for angioectasias was performed for hemostasis but was not successful in our case.

The next option was a combination of estrogen and progesterone, which was refused by the patient. Finally, thalidomide was administered and the patient responded to the medication, which was evident by the resolution of angioectasia during a repeat endoscopy done six months after treatment.

## Introduction

Angioectasias are characterized by a thin-walled, fragile vascular network with a disrupted architecture, increased permeability, and susceptibility to rupture [1-5]. Gastrointestinal (GI) angioectasia is a vascular lesion characterized by vascular ectasias at the submucous sheath of the gastrointestinal tract. Lesions can be flat or raised, isolated or grouped, and can break or ulcerate, causing acute hemorrhage or, more commonly, chronic bleeding [6]. It is responsible for approximately 1.2% to 8.0% of the cases of GI bleed [1-5].

Angioectasias have been reported in association with aortic valve disease, systemic sclerosis, and hereditary hemorrhagic telangiectasia and in patients with end-stage renal disease [7-9].

Here, we present a case of angioectasias in a cirrhotic patient without any aforementioned risk factors for angioectasia who developed upper GI bleeding due to angioectasias of the antrum and duodenum, which is unusual in a cirrhotic patient.

## Case presentation

A 66-year-old male presented to the emergency department (ED) with two episodes of coffee-ground vomiting. He denied any abdominal pain, dizziness, or syncope. He had two episodes of melena in the past with status post-argon plasma coagulation (APC) for prior gastrointestinal angioectasias. Other significant medical comorbidities were chronic alcoholism, cirrhosis, and hypertension (HTN). He had no family history to note. Other than age, he had no specific risk factors for angioectasias; most notably, there was no history of aortic stenosis, von Willebrand disease, or chronic renal failure. His blood pressure (BP) was 115/70 mmHg and heart rate (HR) was 98 beats per minute (bpm) while supine and 93/65 mmHg and 110 bpm while standing. He was afebrile, alert, and oriented to time, person, and location. A physical examination showed multiple spider nevi and a cherry angioma. Abdominal examination was significant for shifting dullness and caput medusae in the abdomen. There was no abdominal tenderness or asterixis. The neurological exam was unremarkable. The hemoglobin level at the time of admission was 6.5 g/dl as compared to the baseline 11 g/dl measured three months ago. He was appropriately resuscitated with intravenous fluid and blood transfusion. Abdominal ultrasound revealed surface nodularity and increased echogenicity with irregular appearing areas consistent with cirrhosis with model of end-stage liver disease (MELD) score 13 and Child Turcotte Pugh score (CTP) 10.

**Figure 1:**
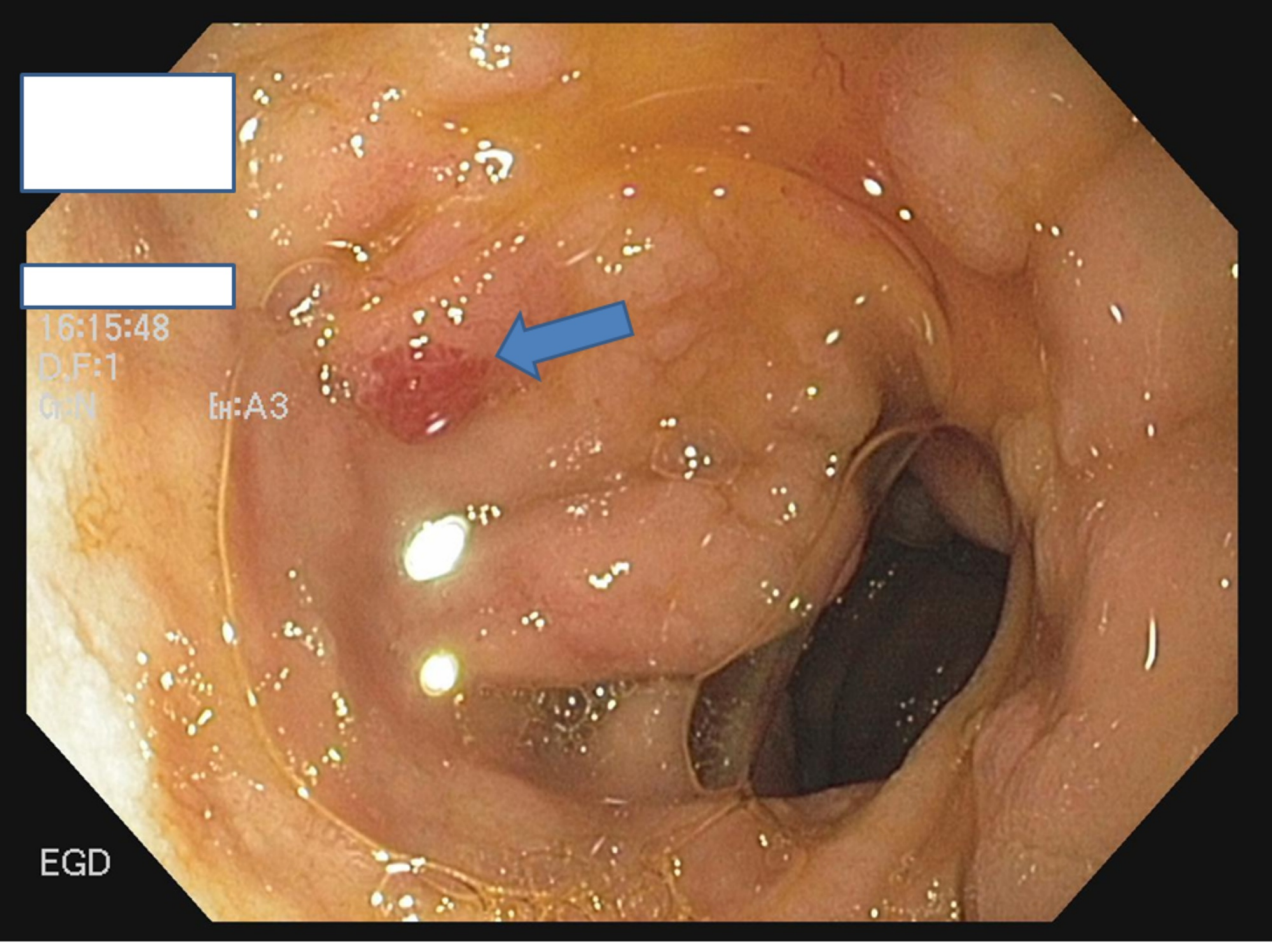
EGD showing angioectasias in the first part of the duodenum (bulb) EGD: esophagogastroduodenoscopy

**Figure 2:**
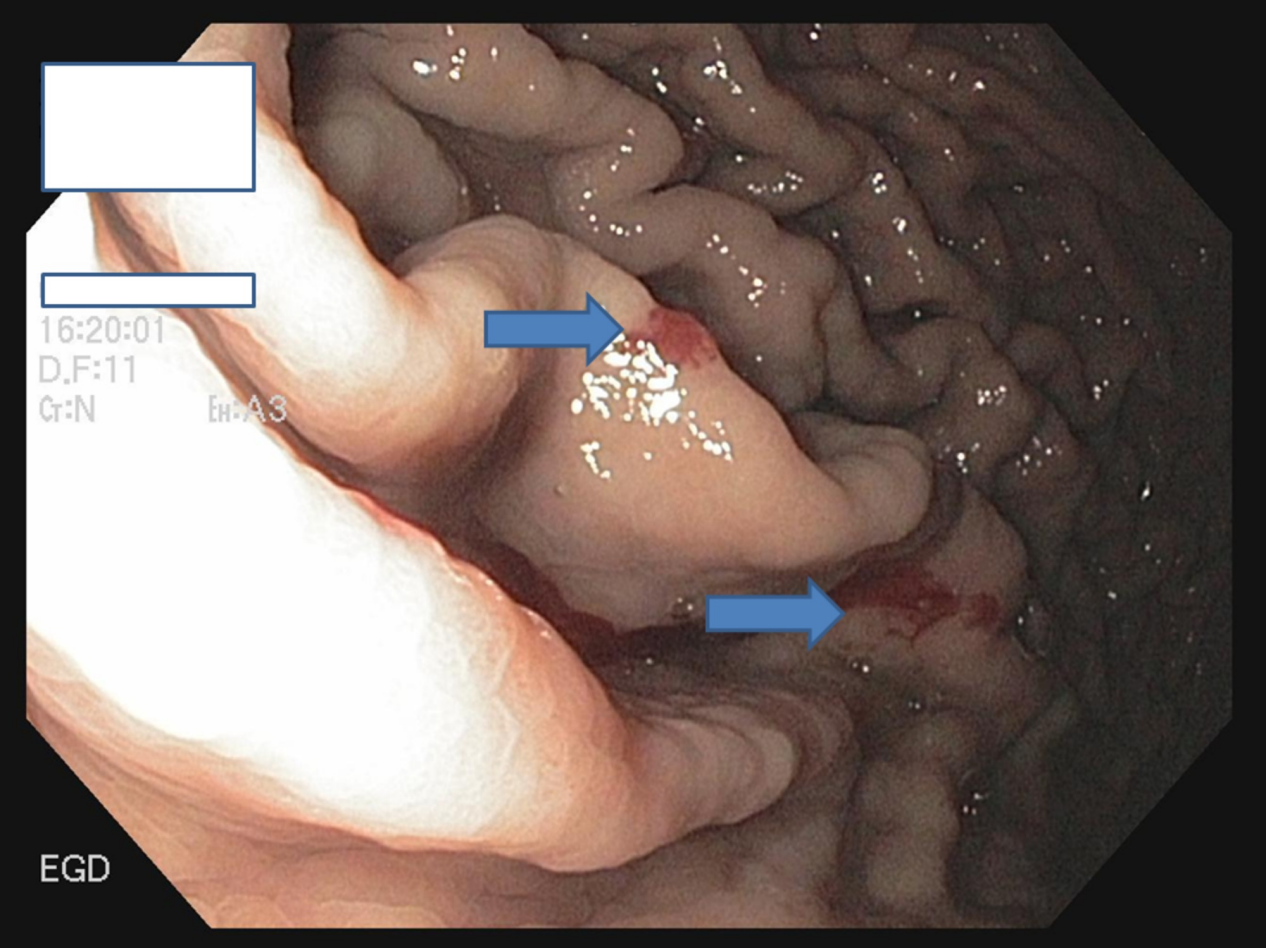
EGD showing multiple angioectasias at the greater curvature of the stomach EGD: esophagogastroduodenoscopy

Esophagogastroduodenoscopy (EGD) was performed and revealed multiple angioectasias in the gastric body, antrum, and first part of the duodenum (Figures 1-2). Since the patient had two earlier sessions of APC in prior admissions without improvement and a recurrence of melena, he opted for medical therapy with thalidomide, as he was not comfortable with estrogen therapy due to the possible adverse effects of estrogen.

**Figure 3:**
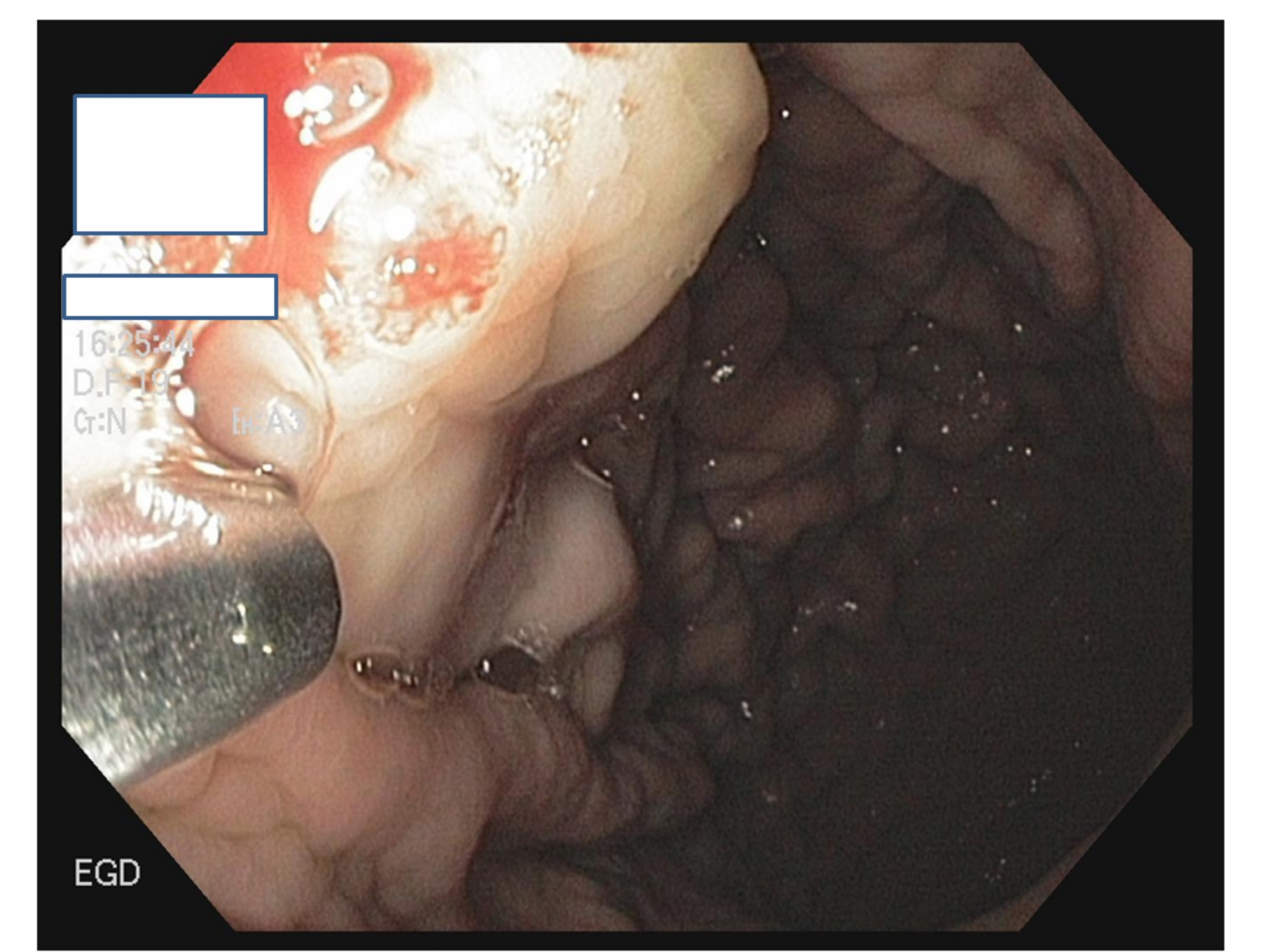
EGD showing APC application at the angiodysplastic lesion EGD: esophagogastroduodenoscopy; APC: argon plasma coagulation

Figure 3 reveals an image from a prior EGD showing the application of APC for angiodysplasia.

**Figure 4:**
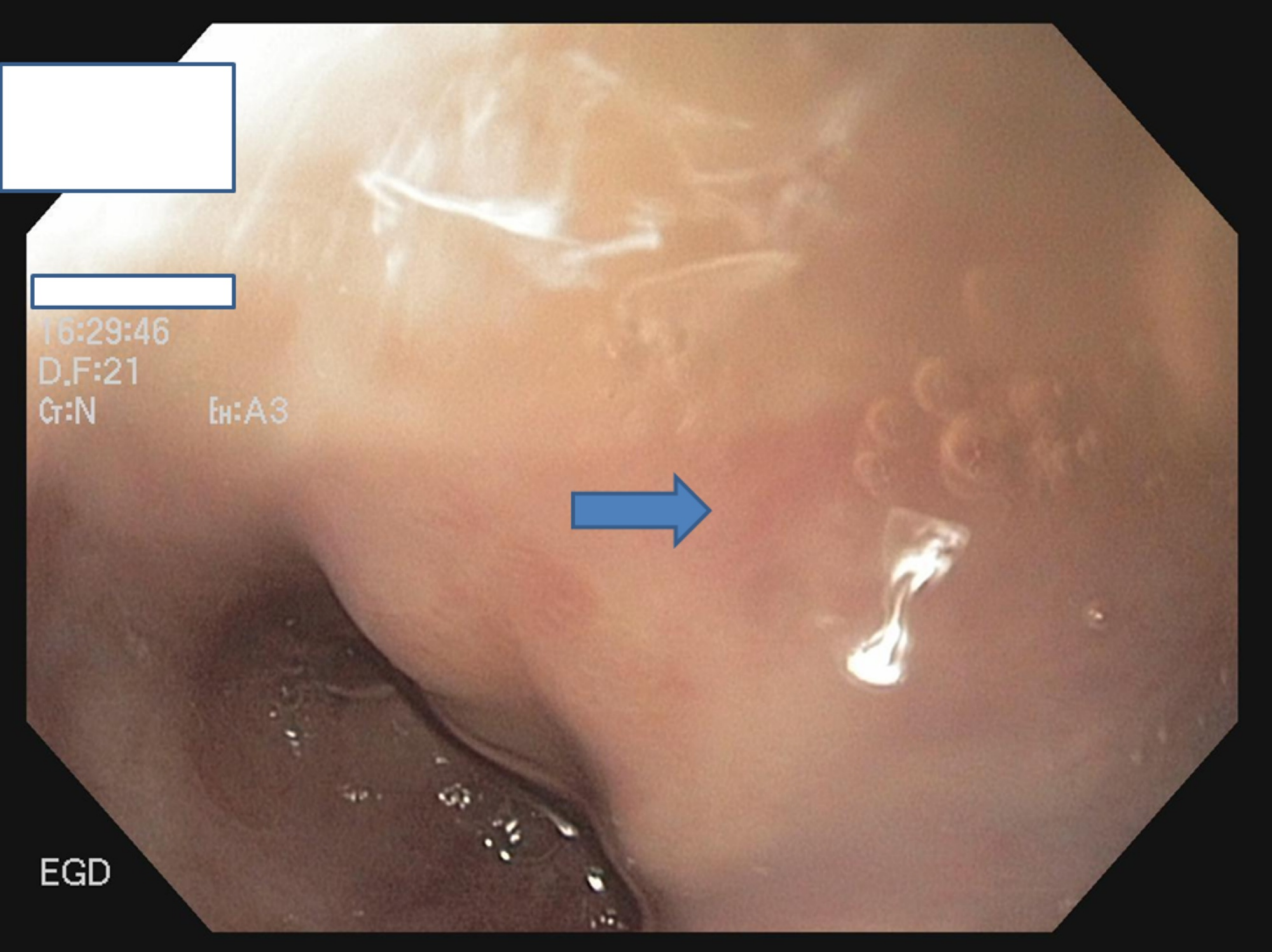
Follow-up EGD at six months showing no recurrence of angioectasias EGD: esophagogastroduodenoscopy

The patient started taking thalidomide 100 mg four times a day. He was treated for a total of six months and EGD showed a marked improvement in angiodysplastic lesions (Figure 4). He was followed for two years after the treatment without a recurrence of GI bleeding.

## Discussion

Variceal bleeding accounts for a high percentage of bleeding episodes overall in individuals with cirrhosis (approximately 80%). Dae Bum et al. reported angiodysplasias in association with aortic valve disease, systemic sclerosis, hereditary hemorrhagic telangiectasia and in patients with end-stage renal disease [10-12].

Our patient did not have any of these associated comorbidities. Etiologies directly related to portal hypertension include esophageal varices, varices, duodenal varices, gastric portal hypertensive gastropathy, and etiologies unrelated to portal hypertension, including coagulopathies and thrombocytopenia, and gastric antral vascular ectasia (GAVE), but scattered angioectasias are not commonly associated with cirrhosis [12]. In our case, EGD ruled out any etiology of upper GI bleeding directly related to portal hypertension, including esophageal, gastric, duodenal varices, and gastric portal hypertensive gastropathy. His coagulation profile and platelet count were within normal limits as well.

Yi-Chun Chiu et al. reported that endoscopic hemostasis with APC is a safe treatment modality for upper gastrointestinal (UGI) angiodysplasia [13]. However, in light of the prior failure of two sessions of APC, medical therapy was preferred by our patient due to the invasive nature of EGD. Harrison et al. reported the use of combination estrogen and progesterone therapy for a variety of arteriovenous malformations (AVMs), including angiodysplasia and hereditary hemorrhagic telangiectasia (HHT), affecting the gut.

The exact mechanism of how hormones improve bleeding from such vessels has not been elucidated. However, a number of hypotheses have been proposed to explain the beneficial effects [14]. These include the stabilization of fragile vessels through the increased keratinization of surrounding squamous epithelium, reduction of leaking from fragile capillaries, improvement of possible coagulation abnormalities, shortening of bleeding time, and vasoconstrictive effect of vasopressin and noradrenaline. Our patient refused to take estrogen for the above-mentioned reasons and he opted for thalidomide, which is an inhibitor of angiogenesis.

There is currently limited evidence for the use of thalidomide in treating angioectasias in patients with liver cirrhosis. One randomized controlled trial (RCT) compared thalidomide 100 mg daily to oral iron in 55 patients refractory to treatment [7]. In this study, the response rate was 71% in the treatment group as compared to 4% in the control group (treatment response defined as at least a 50% reduction in bleeding episodes at one year). Bleeding stopped in 46% of patients receiving thalidomide as compared to 0% in the control group [15].

## Conclusions

Angioectasia is a rare cause of GI bleeding in cirrhosis and can manifest as nonbleeding to overt life‐threatening bleeding. While APC is the gold standard therapy, pharmacologic therapy, such as thalidomide, may be used for the treatment of multiple GI angioectasias in patients with prior failure of APC.
